# Vegetable Contamination by the Fecal Bacteria of Poultry Manure: Case Study of Gardening Sites in Southern Benin

**DOI:** 10.1155/2016/4767453

**Published:** 2016-03-16

**Authors:** Séraphin C. Atidégla, Joël Huat, Euloge K. Agbossou, Hervé Saint-Macary, Romain Glèlè Kakai

**Affiliations:** ^1^Faculté des Sciences Agronomiques, Université d'Abomey-Calavi, 01 BP 526 Cotonou, Benin; ^2^CIRAD UPR HortSys, 34498 Montpellier Cedex 05, France; ^3^CIRAD, UPR Recyclage et Risque, 34398 Montpellier Cedex 05, France

## Abstract

A study was conducted in southern Benin to assess the contamination of vegetables by fecal coliforms,* Escherichia coli*, and fecal streptococci as one consequence of the intensification of vegetable cropping through fertilization with poultry manure. For this purpose, on-farm trials were conducted in 2009 and 2010 at Yodo-Condji and Ayi-Guinnou with three replications and four fertilization treatments including poultry manure and three vegetable crops (leafy eggplant, tomato, and carrot). Sampling, laboratory analyses, and counts of fecal bacteria in the samples were performed in different cropping seasons. Whatever the fertilization treatment, the logs of mean fecal bacteria count per g of fresh vegetables were variable but higher than AFNOR criteria. The counts ranged from 8 to 10 fecal coliforms, from 5 to 8 fecal streptococci, and from 2 to 6* Escherichia coli*, whereas AFNOR criteria are, respectively, 0, 1, and 0. The long traditional use of poultry manure and its use during the study helped obtain this high population of fecal pathogens. Results confirmed that the contamination of vegetables by fecal bacteria is mainly due to the use of poultry manure. The use of properly composted poultry manure with innovative cropping techniques should help reduce the number and incidence of pathogens.

## 1. Introduction

In Sub-Saharan Africa, the products of urban agriculture are considered to be one response to the shortage of foodstuffs [1]. In addition to the contributions of urban agriculture to urban food security, nutrition, and local economies, farming also affects urban water management, sanitation, and health services [2]. In that context, urban production of vegetables is increasing rapidly but, in both Africa and Asia, faces many constraints, especially land pressure, access to water, and low soil fertility [3–5]. In Benin, West Africa, the same problems have been identified in periurban and urban gardening areas, where irrigated vegetable production developed rapidly after 1990, coinciding with the drastic drop in fish resources in the Atlantic Ocean and in the rivers. The main vegetable crops are leafy vegetables, eggplant (*Solanum macrocarpon* L.), carrots (*Daucus carota* L.), tomatoes (*Lycopersicon esculentum* Mill.), peppers (*Capsicum frutescens*), and onions (*Allium cepa*).

To satisfy the increasing demand for vegetables, despite the poverty of coastal soils and land pressure, farmers tend to intensify production by using mineral and organic fertilizers and pesticides. Today, animal manure (60% poultry manure and 40% cattle manure) is frequently used as fertilizer in the study area, Grand-Popo in Benin. Animal manures have been used as effective fertilizers for centuries [6, 7]. Brooks et al. [8] investigated potential microbial runoff associated with the application of poultry litter on the soil. Several other studies pointed to pollution and health risks caused by lack of knowledge and bad practices in the management of livestock manure and chemical fertilizers [1, 3, 9].

Excessive use of fertilizer at each agricultural campaign has been reported in both Africa and Asia, particularly the use of poultry manure at rates of 20 to 50 t·ha^−1^ and the use of mineral fertilizers, such as urea and NPK (10-20-20 nitrogen, phosphorus, and potassium fertilizer), at rates of 1.2 to 2 t·ha^−1^ [6, 9]. Unfortunately, the intensive use of organic matter like cow dung and poultry manure and other animal feces are a significant environmental risk to soils, waters, and crops, including fecal contamination [6].

Many infection outbreaks have been associated with water or food directly or indirectly contaminated by animal manure [10, 11] by identifying* Escherichia coli* and fecal coliforms, which are indicators of fecal pollution [12, 13]. One such example was a major waterborne outbreak of* Escherichia coli* O157:H7 (ECO157) infections with bloody diarrhea and abdominal cramps which lasted from 15 December 1989 to 20 January 1990 in Missouri [14]. Griffin et al. [15] reported the occurrence of many outbreaks of* Escherichia coli* O157:H7 (ECO157) infections in communities, nursing homes, a day care center, and a kindergarten. They mainly took the form of gastrointestinal diseases, bloody diarrhea, hemolytic uremic syndrome, or thrombotic thrombocytopenic purpura. Contaminated manure can contact the product directly when used as a soil fertilizer or indirectly via infiltration of irrigation water or infiltration of water used to wash the product. Ibenyassine et al. [16] and Steele et al. [17] reported that contaminated irrigation water and surface runoff water may be major sources of pathogenic microorganisms that contaminate fruits and vegetables in fields. Animal feces including poultry manure which contain large numbers of bacteria can contaminate croplands and hence agricultural products. Fecal bacteria, including* Escherichia coli*, are responsible for serious outbreaks of diarrhea, particularly in children. Some of these microorganisms, including fecal coliforms,* Escherichia coli*, and fecal streptococci, are life-threatening. Gastrointestinal diseases are ranked as the second most important health problem after malaria for most communities in Accra, Ghana, especially in high-density, low-income areas. Elderly and immune-depressed patients are also exposed to the risk of gastrointestinal problems. Other infectious diseases including hepatitis, typhoid and paratyphoid fever, meningitis, and skin diseases can also be caused by fecal contamination [18–20].

According to the literature, few studies have dealt with the link between the presence of pathogens in freshly harvested vegetables and the wide use of organic material such as poultry manure as fertilizer in market gardening in tropical developing countries [1, 21]. One risk of the intensive use of such organic waste is fecal contamination of the vegetables [3]. Today, poor practices used for the management of livestock manure and chemical fertilizers remain the same as those reported in 2009-2010, since the farmers in the study area have not adopted alternative practices.

In the present study, this issue was addressed by assessing the microbiological quality of vegetables cultivated in market gardening areas in the coastal area of southern Benin. Life-threatening pathogens (fecal coliforms,* Escherichia coli*, and fecal streptococci) were counted both in the poultry manure and in the fresh vegetables after harvest. Both irrigation water and the cultivated soil were analyzed. The main reason why this study focuses on fecal coliforms,* Escherichia coli*, and fecal streptococci is that* Escherichia coli* is an ideal indicator of hygiene in microbiological analyses of raw foods like fresh vegetables. The origin of these fecal pathogens was identified with the aim of recommending ways to reduce these risks.

## 2. Materials and Methods

### 2.1. Study Area

The study was conducted in 2009 and 2010 in the coastal area of southern Benin at market gardening sites in Yodo-Condji (01°46′33′′N-06°10′10′′E, district of Grand-Popo) and Ayi-Guinnou (01°44′36′′N-06°15′48′′E, district of Agoué).

The climate is subequatorial characterized by little variation in temperature (annual average: 27.4°C) and a bimodal rainfall pattern (annual average rainfall: 882 mm). Marine sandy soils, very seeping and porous, make up the two first soils layers (0–18 cm and 18–40 cm) with slightly basic pH between 7.3 and 7.5 [6] at the two sites. The land has been cultivated continuously for several decades without fallow.

Farmers have easy access to groundwater for crop irrigation (through spraying water method) but are limited by the low fertility of the coastal sandy soils. To satisfy growing urban demand and to improve crop productivity, they have adopted intensive practices, such as application of chemical fertilizers combined with high rates of poultry manure, which is available locally. The length of the crop cycle of these vegetables varies from 1.5 to 3.5 months enabling farmers to successively cultivate four vegetable crops per year.

### 2.2. Experimental Design

At each site, the experiment was conducted in a split-plot design with two factors (4 fertilization treatments and 3 vegetable crops) and three replications during four successive vegetable growing periods. Each plot measured 2 m^2^ (2 m × 1 m).

The four growing periods were from 5 May 2009 to 2 September 2009 (period 1) during the long rainy season, from 10 September 2009 to 12 January 2010 (period 2) during the short rainy season and the long dry season, from 20 January 2010 to 13 May 2010 (period 3) during the dry season and the long rainy season, and from 21 May 2010 to 15 August 2010 (period 4) during the long rainy season and the short dry season.

The main factor analyzed was fertilization including the chemical fertilizers and poultry manure applied during each growing period, using four different treatments (Table 1). The poultry manure was composed of chicken feces and wood shavings and came from a local chicken farm.

The second factor was the vegetable crop: tomato (*Lycopersicon esculentum* M.), traditional eggplant (*Solanum macrocarpon* L.), or carrot (*Daucus carota* L.). The vegetables were harvested the same day and only once.

### 2.3. Sampling and Analyses

A total of 164 samples from the two sites were analyzed during the study. At harvest time, for each plot and each crop, five individual samples collected from the four corners of the plot and one sample from the middle were pooled to make a composite 100 g sample of fresh vegetables. The samples were immediately placed in sterile bags. A total of 96 composite samples of fresh vegetables were analyzed for the period of 2009-2010. Eight samples of poultry manure were collected before each sowing date. One kg of poultry manure was collected using the procedure described above. All samples of poultry manure were immediately sealed in sterile bags at 25°C and transported to the laboratory.

Analyses of the different samples (poultry manure and vegetables) were performed by the “Water and Food Quality Control Laboratory of the Ministry of Health” in Cotonou. At the time, this laboratory was the only one in Cotonou to perform microbiological analyses of both liquid and solid foods. During the study period, a second laboratory, which was only qualified for microbiology analyses in water, had problems with its specific equipment. But as soils and poultry manure are solid and the target of the present study was fecal pathogens, samples of soils and poultry manures were also sent to that qualified laboratory. In the absence of evidence from other organizations (FAO, CEE, and IRD) or other European recommendations that mention levels of fecal pathogens recorded in soils and manure, AFNOR food and water criteria are used in the paper. To our knowledge, the purpose of the AFNOR criteria is to identify the concentration of these pathogens in the substances like vegetables or water, consumed by human beings. For this reason, we compare our data concerning soils and poultry manure with AFNOR criteria and analytical techniques.

*(i) Vegetables and Poultry Manure.* Note the following:

- Fecal coliforms per g were identified by colimetry using the V-08-60 Rapid'*E. coli* medium (24 h at 44°C).
- *Escherichia coli* per g were identified by colimetry using the V-08-053 Rapid'*E. coli* medium (24 h at 44°C).
- Fecal streptococci were identified by NFT 90416 and Bartley and Slanetz medium (24 h–48 h at 37°C).

The number of fecal microorganisms per gram of composite sample of fresh vegetables or of poultry manure or of soils was used as the unit of measure.

### 2.4. Data Analysis

Analysis of variance on repeated measures [22] was performed to test the effects of “growing period,” “fertilization treatment,” “vegetable crop,” and “site” on the populations of microorganisms of soils, while traditional analysis of variance (nonrepeated measures) was performed to test the effects of the above-cited factors (except “growing period”) on the populations of fecal microorganisms on the vegetables. In the two models, site was considered as random factor, while the other factors were fixed. These statistical analyses were conducted using SAS software version 9.2.

To stabilize the variances, each of the three variables (fecal coliforms,* Escherichia coli*, and fecal streptococci) was log-transformed according to the following relation: *y* = ln⁡(*x* + 1), where *x* is the number of bacteria observed for each variable and *y* is the result of the transformation.

The adjusted means of the three variables were compiled with the corresponding coefficients of variation. Student-Newman-Keuls (SNK) tests distinguished means by highlighting the different groups of homogenous treatments.

Factors able to explain the variability of the number of fecal bacteria in the study area were identified.

## 3. Results and Discussion

### 3.1. Changes in Populations of Fecal Bacteria in the Poultry Manure as a Function of the Growing Period

**Figure 1:**
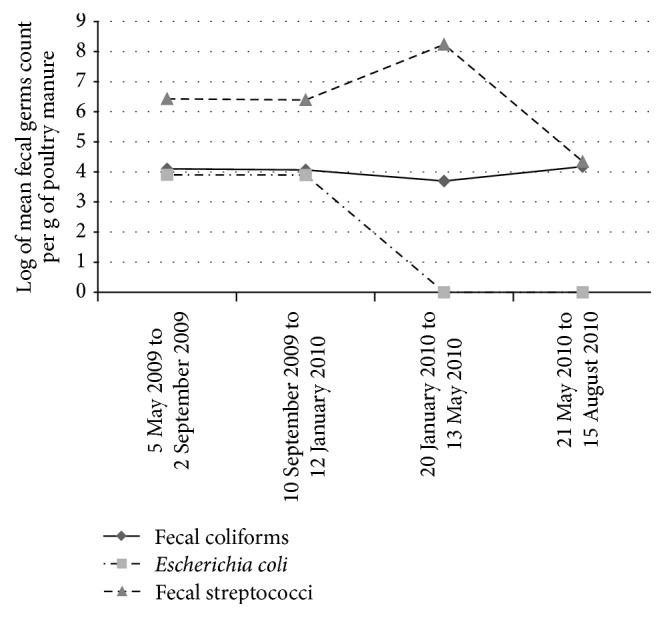
Changes in populations of the three fecal microorganisms in poultry manure over the four growing periods (coastal area of Benin, 2009-2010).

High temporal variability of the number of fecal bacteria in the poultry manure at the two sites was observed (Figure 1). Log-transformed fecal coliform counts increased from 5 May 2009 to 2 September 2009 (period 1) and from 10 September 2009 to 12 January 2010 (period 2). Conversely, log-transformed counts of* Escherichia coli* decreased from 4 in period 1 to slightly less than 4 in period 2 and were absent from 20 January 2010 to 13 May 2010 (period 3) and from 21 May 2010 to 15 August 2010 (period 4). The number of streptococci decreased from more than 6 in period 1 to slightly less than 6 in period 2 and then increased to 8 in period 3 before falling to slightly less than 4 in period 4.

The high temperatures (30°C to 35°C) between December 2009 and January 2010 when the poultry manure was stored outside the henhouse probably helped reduce populations of fecal coliforms by more than half (growing period 2 to growing period 3). A similar situation was observed for* Escherichia coli* in irrigation water and soils at both sites, but here we rather witnessed their disappearance from period 1 (harvest period) to period 3. However, concerning the poultry manure, the untimely actions of the driving rain during the trial could explain the variations that occurred in period 4 when the poultry manure was stored outside the henhouse for two months (from April 2010 to May 2010). Rainfall amounted to 147 mm in eight days from 1 April 2010 to 12 April 2010 versus 16 days for the same volume during the same period in 2009. Driving rains could thus also have been responsible for the reduction in (or the absence of) microorganisms at some dates, in particular* Escherichia coli* and fecal streptococci in the poultry manure taken from the henhouse on 10 April 2010 and analyzed on 13 May 2010. This observation is in accordance with the results of Hutchison et al. [23], who contrasted the effect of driving rain on the survival of fecal coliforms, causing their destruction and washing them out, with the effect of drizzle. Like Jamieson et al. [24], our results showed that bacterial survival was optimal in cold wet conditions. But it is possible that competition among microorganisms also affects the survival of fecal bacteria in the soil, in line with the influence of predation [25]. In the study area, predation among bacteria could also be responsible for the disappearance of fecal pathogens.

Based on the results observed through Figure 1, it is possible to conclude that, because there was no organic matter in the soil, as soon as the poultry manure was applied to the soil, the fecal microorganisms remained on the surface and moved directly towards the vegetables. Franz et al. [26] reported that survival of* Escherichia coli* was optimal in soils rich in organic matter and under flooding. According to these authors, water holding capacity, which depends on soil texture and organic matter content, is known to have an impact on fecal bacteria. Analyses of poultry manure in growing period 4 (the wettest period) revealed the influence of humidity. As soils with a high humus ratio have the highest water holding capacity, they provide a favorable environment for the survival of fecal pathogens. In conclusion, in this study area, a large proportion of the fecal bacteria supplied by the poultry manure did not survive due to unfavorable abiotic conditions (temperature, pH, and organic matter content).

### 3.2. High Level of Contamination of Harvested Vegetables

**Figure 2:**
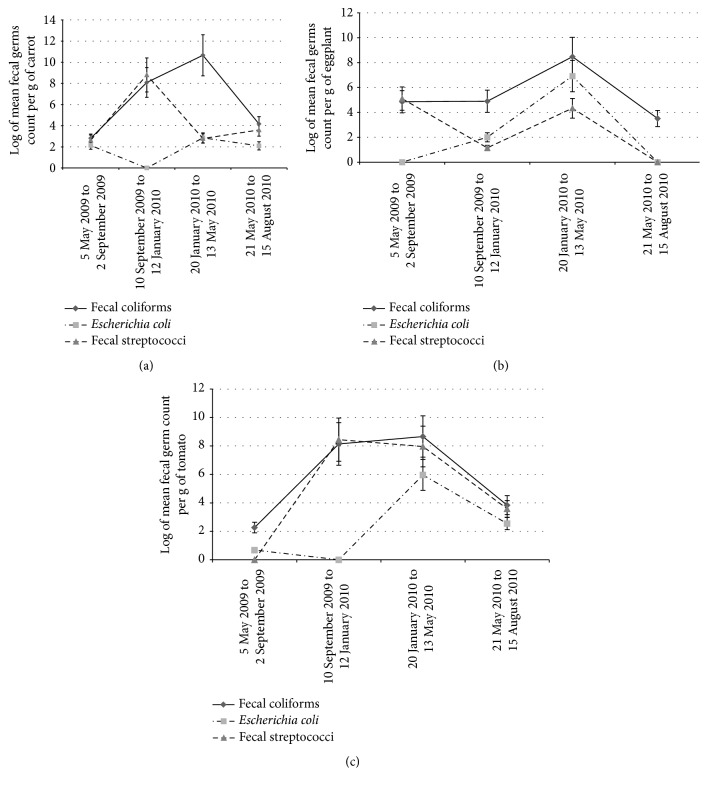
(a) Changes in populations of fecal bacteria on carrot per growing period (sites and fertilization treatments considered together (coastal area of Benin, 2009-2010, 288 plots, 32 samples)). (b) Changes in populations of fecal bacteria on eggplant per growing period (sites and fertilization treatments considered together (coastal area of Benin, 2009-2010, 288 plots, 32 samples)). (c) Changes in populations of fecal bacteria on tomato per growing period (sites and fertilization treatments considered together (coastal area of Benin, 2009-2010, 288 plots, 32 samples)).

**Figure 3:**
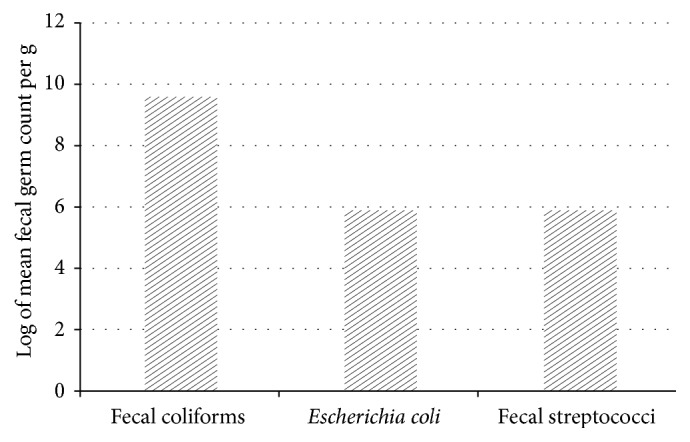
Populations of fecal coliforms,* Escherichia coli*, and fecal streptococci on vegetables (sites, growing periods, fertilization treatments and vegetables considered together (coastal area of Benin, 2009-2010, 288 plots, 96 samples)).

Individually, site, fertilization treatment, and type of vegetable had no significant effect on the number of fecal microorganisms (Figures 2(a), 2(b), and 2(c)). Log-transformed fecal coliform counts on vegetables were close to 10, that is, higher than those of* Escherichia coli* and fecal streptococci (each close to six) (Figure 3).

The number of populations of microorganisms varied considerably among the four growing periods and among the three vegetables crops (Figures 2(a), 2(b), and 2(c)). From 5 May 2009 to 13 May 2010 (three successive growing periods), fecal coliforms increased four times on carrot (2.8 to 10.7) and almost two times on eggplant (4.8 to 8.5). In the same periods, fecal coliforms on tomato increased almost four times (from 2.3 to 8.6) but decreased by half on both vegetables in period 4 (Figures 2(a), 2(b), and 2(c)). On tomato (Figure 2(c)), fecal coliforms increased from two in period 1 to nine in period 3 before decreasing to four in period 4. The highest rate of contamination by fecal coliforms (<11) on carrot and by* Escherichia coli* on eggplant (7) occurred in period 3 and in period 2 on carrot (<9). The number of fecal coliforms on the vegetables reached its peak in periods 2 and 3, whereas the number of* Escherichia coli* (7) was highest in period 3 and that of fecal streptococci (>8) was highest in period 2. Fecal coliforms were lowest (around 2) in period 1.* Escherichia coli* were absent during some growing periods and fecal streptococci were absent in period 4 (Figure 2(b)).

In the vegetable samples, the mean number of fecal bacteria per gram was considerably higher than the levels recommended by the French Standards Association [27] for fresh vegetables (Table 2): 8 to 10 times higher than the standard for fecal coliforms, 2 to 6 times higher than the standard for* Escherichia coli*, and 5 to 8 times higher than the standard for fecal streptococci.

Although the original source of contamination of produce has seldom been identified, manure from farm animals has long been suspected of being a leading vehicle of pathogen transmission. Concerning the different results and the fact that the major source of contamination of fresh products is microbial pathogens, we agree with Ijabadeniyi et al. [11] who reported that, at the preharvest stage, the sources include feces, irrigation water, inadequately composted manure, soil, air, animals, and human handling.

### 3.3. Role of Poultry Manure in the Contamination of Vegetables

**Figure 4:**
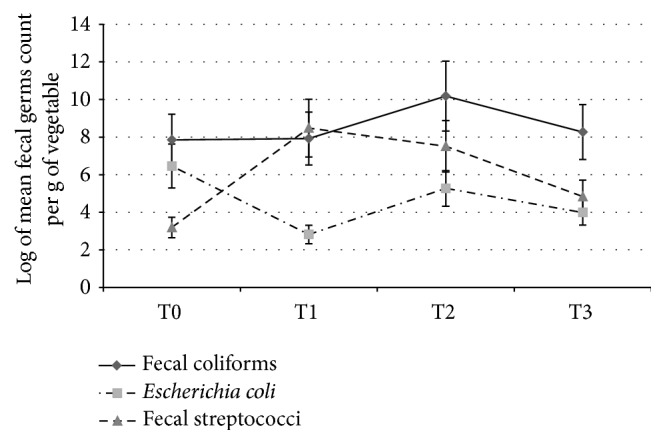
Populations of the three fecal bacteria per fertilization treatment: T0, unfertilized control; T1, mineral fertilization (urea + NPK); T2, mineral fertilization + 10 t·ha^−1^ of poultry manure; and T3, 40 t·ha^−1^ of poultry manure (coastal area of Benin, 2009-2010, 288 plots). The fertilization, with or without poultry manure, had no significant impact on the high initial contamination of the environment.

Considering all the treatments (T0, T1, T2, and T3) together, we found all three types of fecal bacteria in the three vegetable crops (Figures 2(a), 2(b), and 2(c)). As shown by the results of the analyses of variance (Table 3), at *p* < 0.05, only the growing period had a significant effect (probability 0.1%) on the concentrations of the three fecal bacteria analyzed. The application of fertilizer had no significant impact on the high initial contamination of the environment. For instance, the control treatment (T0), that is, no poultry manure, resulted in as high level of fecal coliforms as treatments T1 (mineral fertilizer), T2 (mineral fertilizer + poultry manure), and T3 (manure only), on all three vegetables (Figure 4). We recorded log-transformed average counts of eight for fecal coliforms in the plots with the control treatment T0, six for* Escherichia coli*, and three for fecal streptococci.

On the other hand, treatments T2 and T3 tended to result in more fecal coliforms and fecal streptococci in all the growing periods. This tendency was most marked in period 2 in the short rainy season and in period 3 during the long dry season (Figures 1, 2(a), 2(b), and 2(c)).

The control treatment (T0), under which no poultry manure was applied, resulted in the same high concentration of fecal microorganisms in all three vegetables as treatments T1, T2, and T3. This is likely due to the untimely actions of the flow of irrigation water and rain on the plots under control treatment T0. The long traditional use of poultry manure which probably left fecal bacteria in the soil and water in the study area before the beginning of the trials is another probable explanation.

On the other hand, the lower number of* Escherichia coli* in all three vegetables with treatment T3 confirmed that contamination was recent. Even though three months passed between application of the fertilizer and harvest, it is highly probable that* Escherichia coli* we counted came from the applied poultry manure. So, in the case of Grand-Popo, both traditional organic fertilizers (poultry manure and cow dung) and the poultry manure supplied during the trial can be assumed to be responsible for the contamination. Our results are in agreement with those of Amponsah-Doku et al. [9], who reported that poultry manure, which is the main fertilizer used by 75% of lettuce growers in Accra and Kumasi (Ghana), was responsible for the contamination of the lettuce by numerous fecal coliforms.

Urban areas can also be sources of fecal pollution: storm waters can transport fecal organisms in runoff originating from domestic waste, urban wildlife, or domestic animals. The many heaps of wastes observed in the villages of Ayi-Guinnou and Yodo-Condji and the liquid wastes deposited in the environment along with vegetable leftovers were possible sources of contamination. Moreover, for more than 70% of the local population, river banks and the beach are defecation areas with deleterious consequences for the microbiological quality of the irrigation water, which is also used as drinking water by the local population [1, 28].

## 4. Conclusion

Application of contaminated poultry manure to the crops in gardening sites may result in contamination of vegetables by the following fecal bacteria: fecal coliforms,* Escherichia coli*, and fecal streptococci. Our study has demonstrated that the use of poultry manure as fertilizer during the agronomic trials has influenced the fecal bacteria counts recorded on the vegetables. The long traditional use of poultry manure which probably left fecal bacteria in the soil and water in the study area before the beginning of the trials is another probable explanation.

This statement of the environment constitutes a genuine danger for the public health: that of the farmers, local populations, and the consumers of the vegetables produced in the area. The combination of market gardening and livestock rising is widely considered to be a good way to increase plant yields. But our results show that it can also have negative impacts on human health. For this reason, to achieve sustainable urban agriculture in Africa, those designing innovative cropping systems need to be mindful of the environment and of public health concerns and such systems should be developed through multidisciplinary research that includes more medical, biological, environmental, and socioeconomic components. But, in the immediate future, the use of properly composted poultry manure should help reduce the number and range of pathogens and hence avoid the application of contaminated manure.

## Figures and Tables

**Table 1:** Fertilization modalities at each growing period: application of poultry manure and mineral fertilizers on the three tested vegetable crops (eggplant, tomato, and carrot). Amendment was applied one week before sowing; top-dressing was applied twice: 2 weeks and 4 weeks after sowing (coastal area of Benin, 2009-2010, 288 plots).

Fertilization modalities	Amendment (t·ha^−1^)	Total top-dressing (t·ha^−1^)	Total per growing period (t·ha^−1^)
T0, control	0	0	0
T1, farmer practice 1			
NPK	0.4	0.8	1.2
Urea	0.4	0.8	1.2
T2, farmer practice 2			
NPK	0.4	0.8	1.2
Urea	0.4	0.8	1.2
Poultry manure	10	10	20
T3, poultry manure only	25	15	40

**Table 2:** Populations of fecal coliforms, *Escherichia coli,* and fecal streptococci on each crop (sites, growing periods, and fertilization treatments considered together) in comparison to AFNOR criteria (coastal area of Benin, 2009-2010, 288 plots, 96 samples of all crops).

Log of mean fecal bacteria counted per g of crop	Carrot		Eggplant		Tomato		AFNOR criteria
	M	SD	M	SD	M	SD	
Fecal coliforms	10	6.09	8	5.21	8	5.46	0
*Escherichia coli*	2	1.69	6	2.22	5	2.17	0
Fecal streptococci	8	4.26	5	2.55	8	4.85	1

**Table 3:** Analysis of variance onrepeated measures: Fisher values illustrating the effects of growing period, fertilization modality, vegetable crop, and site on the fecal bacteria populations in vegetables (coastal area of Benin, 2009-2010, 288 plots).

Source	Degree of freedom	*F*_obs_		
		Fecal coliforms (log)	*Escherichia coli* (log)	Fecal streptococci (log)
Growing period	3	87.61^*∗∗∗*^	53.15^*∗∗∗*^	10.94^*∗∗∗*^
Growing period *∗* site	3	1.03^ns^	17.96^*∗∗∗*^	2.25^ns^
Growing period *∗* vegetable	6	2.82^*∗*^	5.73^*∗∗*^	6.59^*∗∗∗*^
Growing period *∗* fertilization	9	1.37^ns^	2.21^ns^	3.32^*∗*^
Growing period *∗* site *∗* vegetable	6	2.18^ns^	3.45^*∗*^	3.55^*∗*^
Growing period *∗* site *∗* fertilization	9	0.74^ns^	4.77^*∗∗*^	1.07^ns^
Growing period *∗* vegetable *∗* fertilization	18	1.48^ns^	1.32^ns^	1.20^ns^

Note: ^*∗*^significant at 0.05; ^*∗∗*^significant at 0.01; ^*∗∗∗*^significant at 0.001; ^ns^not significant at 0.05.
